# Supplementary material for: Factors Associated with Mortality in Ontario Standardbred Racing: 2003–2015
Source: Animals (Basel). 2021 Apr 5;11(4):1028. doi: 10.3390/ani11041028 (PMC8066029; doi:10.3390/ani11041028)
Supplement: Supplementary file 1 [file animals-11-01028-s001.zip › Figure S1.docx]

**Figure S1.**  Logistic regression analysis of mortality data from the Ontario Death Registry for Standardbred horses racing in the Province of Ontario in the period 2003-2015. A. Odds ratios (95% confidence intervals) for mortality (unit of interest - work-event), by SEX and select cumulative career work-events (CMCAR) groups, describing a SEX*CMCAR interaction identified for work-events for which the outcome was SCRATCH. Population of interest - work-events in which horses were scratched (n=65105), response - death registry membership. Referent groups (denominator) are identified on the bottom x-axis, comparator groups (numerators) and significance of comparison are indicated on the top x-axis. Patterns reflect those in Figure 3 for effect of cumulative days raced. A similar pattern identified for COMPLETED work-events did not reach statistical significance. All scratches represent the last race outcome before horses died. * - significant at p=0.05. F - female; G - gelding; S - stallion. B. Probability of mortality (95% confidence intervals, unit of interest - horse-year), by AGE and SEX, describing a significant AGE*SEX interaction. Population of interest - all horse-years for horses racing in the study period (n=125200). When all other factors are held constant, odds rise then falls with age for females, rise continuously for geldings, but begin high, then fall for stallions. F - female; G - gelding; S - stallion.
