# Supplementary material for: Factors Associated with Mortality in Ontario Standardbred Racing: 2003–2015
Source: Animals (Basel). 2021 Apr 5;11(4):1028. doi: 10.3390/ani11041028 (PMC8066029; doi:10.3390/ani11041028)
Supplement: Supplementary file 1 [file animals-11-01028-s001.zip › Figure S2.docx]

**Figure S2**.  Odds of mortality (95% confidence intervals, unit of interest - horse-year, n=125200), by track class (TC) and cumulative days raced in the year (CMD), for Standardbred horses racing in the Province of Ontario in the period 2003-2015, and describing a significant TC*CMD interaction identified through logistic regression analysis of mortality data from the Ontario Death Registry. Odds of mortality fall with increasing CMD at all tracks, but initially fall most rapidly at "B" tracks. Odds are otherwise highest at "C" tracks. Curves for "B" and "C" tracks have been moved to the right by 10 and 20 days, respectively, for clarity. Class "A" - Premier; Class "B" - Signature; Class "C" - Grassroots and Regional. All estimates are significant. * - "A" & "B" significantly different, p = 0.0072: ** - "A" & "B" significantly different, p = 0.0121: ☨ - "C" significantly different from "A" & “B”, p ≤ 0.0052.
