# Supplementary material for: Factors Associated with Mortality in Ontario Standardbred Racing: 2003–2015
Source: Animals (Basel). 2021 Apr 5;11(4):1028. doi: 10.3390/ani11041028 (PMC8066029; doi:10.3390/ani11041028)
Supplement: Supplementary file 1 [file animals-11-01028-s001.zip › Table S1.docx]

| **Table S1.** Distribution of Mortality Rates According to Age, Presenting Complaint and Outcome Group for Mortalities in Standardbred Horses in the Ontario Racing Death Registry for the period 2003-2015. (Data presented graphically in Figure 1.) | | | | | | | | | | |
| --- | --- | --- | --- | --- | --- | --- | --- | --- | --- | --- |
|  |  | Presenting Complaint | | | | | | | | |
| AGE |  | Musculo-skeletal | Died Suddenly | Colic | Medical | Iatrogenic | Accidents | Neurological | Hemorrhage | Unknown |
|  |  | COMPLETED* | | | | | | | | |
|  |  |  |  |  |  |  |  |  |  |  |
| 2 |  | 0.1176 | 0.0882 | 0.0686 | 0.0784 | 0.0392 | 0.0196 | 0.0196 | 0.0098 | 0 |
| 3 |  | 0.0872 | 0.0449 | 0.0740 | 0.0581 | 0.0370 | 0.0344 | 0.0238 | 0 | 0.0026 |
| 4 |  | 0.0739 | 0.0433 | 0.0382 | 0.0433 | 0.0255 | 0.0204 | 0.0280 | 0.0025 | 0 |
| 5 |  | 0.0623 | 0.0415 | 0.0692 | 0.0277 | 0.0346 | 0.0242 | 0.0035 | 0 | 0 |
| 6 |  | 0.1084 | 0.0444 | 0.0986 | 0.0394 | 0.0296 | 0.0246 | 0.0148 | 0 | 0 |
| 7 |  | 0.1003 | 0.0358 | 0.0430 | 0.0645 | 0.0215 | 0.0215 | 0 | 0 | 0 |
| 8 |  | 0.1235 | 0.1123 | 0.1011 | 0.0449 | 0.0898 | 0.0337 | 0 | 0 | 0 |
| 9 |  | 0.1510 | 0.0755 | 0.0377 | 0.0189 | 0.0944 | 0 | 0 | 0 | 0 |
| 10 |  | 0.0972 | 0.0972 | 0.1296 | 0 | 0.0324 | 0 | 0 | 0 | 0 |
| Mean |  | 0.0920 | 0.0545 | 0.0686 | 0.0451 | 0.0357 | 0.0240 | 0.0152 | 0.0012 | 0.0006 |
| n |  | 157 | 93 | 117 | 77 | 61 | 41 | 26 | 2 | 1 |
|  |  |  |  |  |  |  |  |  |  |  |
|  |  | DNF* | | | | | | | | |
|  |  |  |  |  |  |  |  |  |  |  |
| 2 |  | 22.9358 | 2.2936 | 0 | 0 | 0 | 0 | 0 | 0 | 0 |
| 3 |  | 17.3378 | 2.7964 | 0 | 0 | 1.1186 | 2.2371 | 0.5593 | 0 | 0 |
| 4 |  | 15.6760 | 6.5317 | 0 | 0 | 0 | 1.9595 | 0 | 0 | 0 |
| 5 |  | 12.1043 | 1.8622 | 0 | 0.9311 | 0 | 0.9311 | 0 | 0 | 0 |
| 6 |  | 19.4444 | 6.9444 | 0 | 1.3889 | 0 | 2.7778 | 1.3889 | 0 | 0 |
| 7 |  | 30.6122 | 10.2041 | 0 | 0 | 0 | 2.0408 | 0 | 0 | 0 |
| 8 |  | 64.4068 | 3.3898 | 3.3898 | 3.3898 | 0 | 0 | 0 | 0 | 0 |
| 9 |  | 58.2011 | 5.2910 | 0 | 0 | 0 | 5.2910 | 0 | 0 | 0 |
| 10 |  | 100.0000 | 20.0000 | 0 | 0 | 0 | 10.0000 | 0 | 0 | 0 |
| Mean |  | 22.7475 | 4.9063 | 0.1487 | 0.4460 | 0.2974 | 1.9328 | 0.2974 | 0 | 0 |
| n |  | 153 | 33 | 1 | 3 | 2 | 13 | 2 | 0 | 0 |
|  |  |  |  |  |  |  |  |  |  |  |
|  |  | SCRATCH* | | | | | | | | |
|  |  |  |  |  |  |  |  |  |  |  |
| 2 |  | 0.6227 | 0.6227 | 0.9340 | 0 | 0.3113 | 0 | 0 | 0 | 0 |
| 3 |  | 0.6236 | 0.7795 | 0.4677 | 0.8574 | 0.0779 | 0.4677 | 0.0779 | 0.0779 | 0 |
| 4 |  | 0.3393 | 0.4071 | 0.4071 | 0.4750 | 0.4071 | 0.5429 | 0.1357 | 0.0679 | 0 |
| 5 |  | 0.3537 | 0.5306 | 0.5306 | 0.4422 | 0.6190 | 0 | 0.0884 | 0 | 0 |
| 6 |  | 0.4888 | 0.1222 | 1.0997 | 0.4888 | 0.4888 | 0.3666 | 0 | 0.1222 | 0 |
| 7 |  | 0.6987 | 0.5240 | 0.3493 | 0.6987 | 0.6987 | 0 | 0 | 0 | 0 |
| 8 |  | 1.3148 | 0 | 0.2630 | 0.5259 | 0.7889 | 0.2630 | 0 | 0 | 0 |
| 9 |  | 0.4139 | 0.4139 | 0.8278 | 0.4139 | 0 | 1.2417 | 0 | 0 | 0 |
| 10 |  | 1.3774 | 0 | 0.6887 | 0.6887 | 0 | 0 | 0 | 0 | 0 |
| Mean |  | 0.5530 | 0.4454 | 0.5530 | 0.5530 | 0.3994 | 0.3226 | 0.0614 | 0.0461 | 0 |
| n |  | 36 | 29 | 36 | 36 | 26 | 21 | 4 | 3 | 0 |
|  |  |  |  |  |  |  |  |  |  |  |
|  |  |  |  |  |  |  |  |  |  |  |
| * Mortality Rate /1000 Events | | | |  |  |  |  |  |  |  |
