# Supplementary material for: Factors Associated with Mortality in Ontario Standardbred Racing: 2003–2015
Source: Animals (Basel). 2021 Apr 5;11(4):1028. doi: 10.3390/ani11041028 (PMC8066029; doi:10.3390/ani11041028)
Supplement: Supplementary file 1 [file animals-11-01028-s001.zip › Table S2.docx]

| **Table S2.** Results of Logistic Regression Modelling of Associations with Membership in the Ontario Death Registry (binary response) for All Standardbred Racehorse Work-events, 2003-2015 - Full Population Mortality Model. | | | | | | |
| --- | --- | --- | --- | --- | --- | --- |
| Events |  | 1778330 |  |  |  |  |
| Registry cases |  | 973 |  |  |  |  |
| Variable |  | Estimate | s.e. | p-value | OR | 95%CI |
| Intercept |  | -5.8866 | 0.8712 | <0.0001 |  |  |
| AGE (years, 4.92) |  | -0.1765 | 0.0728 | 0.02 | 0.838 | 0.727 - 0.967 |
| SEX (F vs S) |  | -1.5330 | 0.2420 | <0.0001 | 0.216 | 0.134 - 0.347 |
| SEX (G vs S) |  | -2.1982 | 0.2250 | <0.0001 | 0.111 | 0.071 - 0.173 |
| GAIT (P vs T) |  | 0.0252 | 0.2584 | 0.3 | 1.026 | 0.618 - 1.702 |
| START (N vs Y) |  | 0.8535 | 0.7430 | 0.05 | 2.348 | 0.547 - 10.072 |
| OUTC (1-5 vs 9-17) |  | -0.5552 | 0.8718 | 0.5 | 0.574 | 0.104 - 3.169 |
| OUTC (6-7 vs 9-17) |  | -0.5012 | 0.8995 | 0.6 | 0.606 | 0.104 - 3.532 |
| OUTC (8 vs 9-17) |  | 0.6657 | 0.9611 | 0.5 | 1.946 | 0.296 - 12.800 |
| OUTC (DNF vs 9-17) |  | 2.2896 | 0.8979 | 0.01 | 9.871 | 1.698 - 57.368 |
| OUTC (SCR vs 9-17) |  | 1.1066 | 1.1248 | 0.3 | 3.024 | 0.334 - 27.418 |
| YEAR (5.27) |  | 0.0506 | 0.0447 | 0.3 | 1.052 | 0.964 - 1.148 |
| TC (A vs C) |  | 0.0019 | 0.7493 | 1.0 | 1.002 | 0.231 - 4.352 |
| TC (B vs C) |  | 0.2533 | 0.7275 | 0.7 | 1.288 | 0.31 - 5.361 |
| YD (/30, 6.33) |  | 0.0330 | 0.0180 | 0.09 | 1.034 | 0.998 - 1.071 |
| CMYR (/10, 1.196) |  | -0.1890 | 0.1000 | 0.06 | 0.828 | 0.680 - 1.007 |
| GAIT*OUTC (P, DNF) * |  | 0.6852 | 0.3197 | 0.03 | 1.984 | 1.060 - 3.713 |
| START*OUTC (N, DNF) * |  | -1.8593 | 0.7663 | 0.02 | 0.156 | 0.035 - 0.699 |
| AGE*SEX (F vs S) * |  | 0.1469 | 0.0513 | 0.004 | 1.158 | 1.047 - 1.281 |
| AGE*SEX (G vs S) * |  | 0.2666 | 0.0443 | <0.0001 | 1.306 | 1.197 - 1.424 |
| YEAR*START (N vs Y) * |  | -0.0755 | 0.0319 | 0.02 | 0.927 | 0.871 - 0.987 |
| AGE*OUTC (DNF vs 9-17) * |  | 0.1607 | 0.0645 | 0.01 | 1.174 | 1.035 - 1.333 |
| AGE*CMYR |  | 0.0390 | 0.0160 | 0.01 |  |  |
| AGE*YEAR |  | -0.0092 | 0.0041 | 0.03 |  |  |
| YEAR*YD (/30) |  | -0.0060 | 0.0030 | 0.01 |  |  |
| * Ratio of odds ratios; OR - odds ratio; GAIT - P-Pacer, T-Trotter; SEX - F - female, G - gelding, S - stallion; YEAR - calendar year, 2003 - 2015; AGE in years; START - N (qualifier or schooling race), Y (race start); TC - track class, A - Premier, B Signature, C - Grassroots and Regional; OUTC - finish position, 1-5 - finished in the first 5; 6-7 - finished 6th or 7th; 8 - finished 8th; 9-17 - finished 9th to 17th; DNF - Did Not Finish; CMYR - cumulative work-events for the current year, in increments of 10; CMCAR - cumulative work-events for career to current year, in increments of 10; YD - Yearday - day of the year, 1-366, in increments of 30; N.A. - not applicable; n/s - not significant. The table shows results significant at p<0.05 and for variables involved in significant interactions. Referents for categorical variables and means for continuous variables are underlined. | | | | | | |
