# Supplementary material for: Factors Associated with Mortality in Ontario Standardbred Racing: 2003–2015
Source: Animals (Basel). 2021 Apr 5;11(4):1028. doi: 10.3390/ani11041028 (PMC8066029; doi:10.3390/ani11041028)
Supplement: Supplementary file 1 [file animals-11-01028-s001.zip › Table S3.docx]

| **Table S3**. Distribution of Mortality Rates According to Presenting Complaint and AGE, stratified by SEX for Mortalities in Standardbred Horses in the Ontario Racing Death Registry for the period 2003-2015. (Presented graphically in Figure 2B.) | | | | | | | | | | |
| --- | --- | --- | --- | --- | --- | --- | --- | --- | --- | --- |
|  |  |  |  |  |  |  |  |  |  |  |
|  |  | Presenting Complaint | | | | | | | | |
| AGE |  | Musculo-skeletal | Died Suddenly | Colic | Medical | Iatrogenic | Accidents | Neurological | Hemorrhage | Unknown |
|  |  |  |  |  |  |  |  |  |  |  |
|  |  | STALLION* | | | | | | | | |
|  |  |  |  |  |  |  |  |  |  |  |
| 2 |  | 0.9838 | 0.3514 | 0.3514 | 0.2811 | 0.1405 | 0.0703 | 0.1405 | 0.0703 | 0 |
| 3 |  | 0.5451 | 0.3115 | 0.1557 | 0.2336 | 0.1298 | 0.1298 | 0.0779 | 0 | 0 |
| 4 |  | 0.2614 | 0.0871 | 0.2033 | 0.2033 | 0.1162 | 0.0581 | 0.0290 | 0 | 0 |
| 5 |  | 0.1536 | 0.0768 | 0.0768 | 0.0384 | 0.0768 | 0.0384 | 0 | 0 | 0 |
| 6 |  | 0.2705 | 0 | 0.2164 | 0.1082 | 0.1082 | 0.0541 | 0 | 0 | 0 |
| 7 |  | 0.3857 | 0.2314 | 0.0771 | 0.1543 | 0.0771 | 0 | 0 | 0 | 0 |
| 8 |  | 0.8387 | 0.3595 | 0 | 0 | 0.1198 | 0 | 0 | 0 | 0 |
| 9 |  | 0.5568 | 0.1856 | 0 | 0.1856 | 0 | 0 | 0 | 0 | 0 |
| 10 |  | 0.6506 | 0.3253 | 0.3253 | 0 | 0 | 0 | 0 | 0 | 0 |
|  |  |  |  |  |  |  |  |  |  |  |
|  |  | FEMALE* | | | | | | | | |
|  |  |  |  |  |  |  |  |  |  |  |
| 2 |  | 0.1776 | 0.1184 | 0 | 0 | 0 | 0 | 0 | 0 | 0 |
| 3 |  | 0.1744 | 0.0619 | 0 | 0 | 0.0506 | 0.0506 | 0.0338 | 0 | 0 |
| 4 |  | 0.0957 | 0.0837 | 0 | 0 | 0 | 0.0538 | 0 | 0 | 0 |
| 5 |  | 0.0998 | 0.0635 | 0 | 0.0272 | 0 | 0.0454 | 0 | 0 | 0 |
| 6 |  | 0.1745 | 0.0582 | 0 | 0.0436 | 0 | 0.0436 | 0.0145 | 0 | 0 |
| 7 |  | 0.2145 | 0.1430 | 0 | 0 | 0 | 0.0715 | 0 | 0 | 0 |
| 8 |  | 0.2277 | 0.0911 | 0.0455 | 0.1366 | 0 | 0 | 0 | 0 | 0 |
| 9 |  | 0.2831 | 0 | 0 | 0 | 0 | 0.0944 | 0 | 0 | 0 |
| 10 |  | 0.4479 | 0.2240 | 0 | 0 | 0 | 0 | 0 | 0 | 0 |
|  |  |  |  |  |  |  |  |  |  |  |
|  |  | GELDING* | | | | | | | | |
|  |  |  |  |  |  |  |  |  |  |  |
| 2 |  | 0.0245 | 0.0245 | 0.0245 | 0 | 0.0245 | 0 | 0 | 0 | 0 |
| 3 |  | 0.1131 | 0.0509 | 0.0566 | 0.0509 | 0.0170 | 0.0509 | 0.0113 | 0 | 0 |
| 4 |  | 0.1594 | 0.0773 | 0.0531 | 0.0241 | 0.0241 | 0.0386 | 0.0435 | 0.0097 | 0 |
| 5 |  | 0.1211 | 0.0666 | 0.0908 | 0.0605 | 0.0545 | 0 | 0.0061 | 0 | 0 |
| 6 |  | 0.1846 | 0.0883 | 0.1445 | 0.0642 | 0.0562 | 0.0482 | 0 | 0 | 0 |
| 7 |  | 0.2090 | 0.0440 | 0.0550 | 0.0990 | 0.0440 | 0 | 0 | 0 | 0 |
| 8 |  | 0.3660 | 0.0955 | 0.1591 | 0.0636 | 0.1432 | 0.0318 | 0 | 0 | 0 |
| 9 |  | 0.3534 | 0.1262 | 0.1010 | 0 | 0 | 0.0757 | 0 | 0 | 0 |
| 10 |  | 0.4420 | 0.1205 | 0.1607 | 0.0402 | 0 | 0 | 0 | 0 | 0 |
|  |  |  |  |  |  |  |  |  |  |  |
|  |  |  |  |  |  |  |  |  |  |  |
|  |  |  |  |  |  |  |  |  |  |  |
|  |  |  |  |  |  |  |  |  |  |  |
| * Mortality Rate /1000 Events | | | |  |  |  |  |  |  |  |
|  |  |  |  |  |  |  |  |  |  |  |
