# Supplementary material for: Factors Associated with Mortality in Ontario Standardbred Racing: 2003–2015
Source: Animals (Basel). 2021 Apr 5;11(4):1028. doi: 10.3390/ani11041028 (PMC8066029; doi:10.3390/ani11041028)
Supplement: Supplementary file 1 [file animals-11-01028-s001.zip › Table S5.docx]

| **Table S5. R**esults of Logistic Regression Modelling of Associations with Failure to Finish a Work-event (DNF, binary response) for Ontario Standardbred Horses for the Period 2003-2015 - All DNF Model, unit of interest - work-event. Only started work-events, no scratches. | | | | |
| --- | --- | --- | --- | --- |
| Unit of interest - work-event |  |  |  |  |
| Outcome - DNF |  |  |  |  |
| Total Work-events - |  | 1712699 |  |  |
| DNF outcomes - |  | 6721 |  |  |
| Variable |  | Estimate | s.e. | p-value * |
|  |  |  |  |  |
| Intercept |  | -4.7614 | 0.3251 | <.0001 |
| AGE (years, 4.91) |  | -0.0392 | 0.0170 | 0.02 |
| START, N *vs*. Y |  | 1.2194 | 0.4437 | 0.006 |
| TC, A *vs*. C |  | -1.8532 | 0.4505 | <.0001 |
| TC, B *vs*. C |  | -0.4438 | 0.3261 | 0.2 |
| YEAR (5.28) |  | -0.0421 | 0.0102 | <.0001 |
| CMYR (/10, 1.19) |  | -0.2344 | 0.0394 | <.0001 |
| CMCAR (/10, 4.37) |  | -0.0442 | 0.0053 | <.0001 |
| CMD (/10, 12.38) |  | 0.0418 | 0.0063 | <.0001 |
| PPOSN (4.62) |  | -0.0290 | 0.0108 | 0.008 |
| GAIT, P *vs*. T |  | -0.2482 | 0.3373 | 0.5 |
| SEX, F *vs*. S |  | -0.2729 | 0.3332 | 0.4 |
| SEX, G *vs*. S |  | -0.0563 | 0.3229 | 0.9 |
| Yearday (/30, 6.33) |  | -0.0767 | 0.0221 | 0.0007 |
| Yearday*Yearday |  | 0.0049 | 0.0014 | 0.0006 |
| CMD*CMD |  | -0.0010 | 0.0002 | <.0001 |
| YEAR*GAIT, P *vs*. T |  | 0.0259 | 0.0075 | 0.0006 |
| CMYR*START, N *vs*. Y |  | 0.7102 | 0.0663 | <.0001 |
| CMD*START, N *vs*. Y |  | -0.0389 | 0.0058 | <.0001 |
| YEAR*AGE |  | 0.0073 | 0.0019 | 0.0001 |
| PPOSN*GAIT, T *vs*. P |  | -0.0290 | 0.0108 | 0.008 |
| PPOSN*START, N *vs.* Y |  | -0.0290 | 0.0108 | 0.008 |
| GAIT*TC*START, P-A-N |  | -2.3798 | 0.6952 | 0.0006 |
| SEX*GAIT*TC, F-P-A |  | -1.6176 | 0.5529 | 0.003 |
| SEX*GAIT*TC*START, F-P-A-N |  | 2.2820 | 0.7850 | 0.004 |
|  |  |  |  |  |
| * Significance was set at 0.01 for this model. OR - odds ratio; CI - confidence interval; GAIT - P-Pacer, T-Trotter; SEX - F - female, G - gelding, S - stallion; START - N - qualifier or schooling race, Y - race start; YEAR - calendar year, 0-12 (2003-2015); AGE - age in years; PPOSN - post position; TC - track class, A - Premier, B Signature, C - Grassroots and Regional; CMYR - cumulative work-events for the current year, in increments of 10; CMD - cumulative days of racing for the current year, in increments of 10; CMCAR - cumulative work-events for career to current work-event, in increments of 10; YD - Yearday - day of the year, 1-366, in increments of 30. The table shows results significant at p<0.01. Referents are underlined. No differentiation was made between work-events for cases in the registry and those not members of the registry. Death registry membership was not included as a variable. Referents for categorical variables and means for continuous variables are underlined. | | | | |
